# *Ex Vivo* Study of Laban's Role in Decreasing Hemolysis Crisis in G6PD-Deficient Patients

**DOI:** 10.1155/2020/8034672

**Published:** 2020-01-21

**Authors:** Wissam Zam, Loay Belal

**Affiliations:** ^1^Al-Andalus University for Medical Sciences, Department of Analytical and Food Chemistry, Tartous, Syria; ^2^Tartous University, Department of Analytical and Food Chemistry, Tartous, Syria

## Abstract

In spite of the vast nutritional and environmental benefits provided by fava bean (*Vicia faba*), the ingestion of vicine/convicine provokes an acute hemolytic anemia called favism in individuals with a glucose-6-phosphate dehydrogenase (G6PD) deficiency. The elimination of these glycosides is a goal that could be accomplished using different processing methods including bacteriological treatment. Laban as a good source of lactic acid bacteria was tested in an *ex vivo* assay on human blood samples in order to determine its capacity in decreasing the hemolysis crisis induced by the ingestion of fava beans. Results indicate a significant decrease in human blood cell hemolysis after the treatment of fava beans by Laban. This decrease in hemolysis was also correlated with the G6PD deficiency categorization. The highest hemolysis level (mean: 23.11 ± 0.76%) was observed in samples with G6PD activity between 10 and 30%, while the lowest hemolysis level (mean: 5.75 ± 0.64%) was observed in samples with G6PD activity more than 60%. This decrease was correlated with a high antioxidant capacity of Laban (51.61 ± 1.13% expressed by the percentage inhibition of DPPH radical). The counts of isolates from MRS and M17 culture plates were 6.75 ± 0.095 and 7.91 ± 0.061 log cfu ml^–1^, respectively. In conclusion, the synergy between the antioxidant properties of Laban and the possible decrease of vicine and convicine concentrations by lactobacillus found in the fermented dairy products could explain the ability of Laban to reduce the hemolysis crisis *ex vivo*.

## 1. Introduction

Fava bean (*Vicia faba*) is a popular crop grown in different climates all around Asia, Europe, and Africa [1]. It is an important source of protein and various nutrients such as phytonutrients, dietary fiber, vitamins, and minerals [2]. In spite of these advantages, fava beans contain some antinutritional compounds including two glycosidic aminopyrimidine derivatives, vicine and convicine, which accumulate at maturity and are metabolized into isouramil and divicine upon hydrolysis [3]. These highly redox proteins rapidly oxidize NADPH and glutathione which are normally detoxified by catalase and glutathione peroxidase, in enzymatic reactions that depend on NADPH [4]. Hence, in favism, a disease that results from a deficiency of the enzyme glucose-6-phosphate dehydrogenase (G6PD), erythrocytes are susceptible to oxidant stress due to poor antioxidant red cell ability and depletion of glutathione peroxidase and catalase [5].

Due to its high nutritional value and importance, several studies have been conducted to reduce the concentration of vicine and convicine from fava beans using different processing methods such as roasting and boiling [6]. Bacteriological treatment was also proved to be one of the most effective methods since *β*-glucosidase is widespread within lactic acid bacteria, but the level of expression is largely dependent on the strain [1, 7]. Laban is a popular food with a high nutritional value consumed in different areas. It is prepared by the fermentation of milk by lactic acid bacteria [8]. It is found to be useful in the treatment of multiple diseases such as obesity, cancer, and diabetes [9]. Bovine milk used in the preparation of Laban contains a wide variety of compounds with antioxidant activity such as flavonoids and vitamin E [8, 9]. Furthermore, Laban contains lactic acid bacteria that were proved to have a *β*-glycosidase activity that hydrolyzes the glycoside bond [1, 10].

To our knowledge, this is the first study to investigate, through the *ex vivo* assays on human blood, the effect of Laban in decreasing the hemolysis crisis in patients with G6PD deficiency.

## 2. Materials and Methods

### 2.1. Fava Bean Extract (FBE)

Fava beans grown in Tartous, Syria, were collected fresh after maturity and kept at –18°C until use. The sample was extracted according to Sosulski and Pitz with slight modification [11]. Ground seed samples were extracted in duplicate with 0.1N NaOH for 20 minutes. The pH was adjusted to pH 4.2 by HCl 1N in order to precipitate most of the proteins in their isoelectric pH. Samples were centrifuged for 20 minutes at 10000 rpm, and supernatants were collected for further experiments. A blank was prepared for the spectrophotometric measurement.

### 2.2. Fava Beans Treated by Laban (FBL)

Syrian fermented milk (Laban) was prepared using bovine milk one day before use. It was made by boiling the milk for 5 min, permitting it to cool to 50°C, and inoculating it for about 4 hr with 2.5 to 3.0% starter saved from a previous batch.

Ground seed samples of fava beans were incubated with Laban at a ratio of 1 : 2 for 30 minutes. Then, the sample was extracted as described above. A blank was also prepared for the spectrophotometric measurement.

### 2.3. Human Blood Hemolysis

This study was designed to evaluate the *in vitro* effect of Laban on the blood hemolysis caused by fava beans in children aged between 5 and 12. Before drawing the blood samples, parents of all participants were counseled and those who consented (written informed consent) to their children being part of the study were recruited.

Blood samples from children were drawn on EDTA tubes and used for the screening of the subjects for G6PD deficiency using fluorescent spot test (FST) [12]. Children with red blood cell G6PD value of <6.40 U/gHb were regarded as deficient and were recruited.

The determination of the human blood hemolysis was performed with appropriate local health regulations and ethical approval as described by Rizzello et al. [3]. Fully dissolved blood samples were used to determine maximum degeneration using purified water. 0.5 ml of each blood sample was incubated with 2.5 ml from each of FBE, FBL, and blanks for 5 min and then centrifuged to separate the cells from the plasma. An aliquot of plasma was diluted with Drabkin's reagent (leading to the conversion of hemoglobin to cyanmethemoglobin), and the OD540 was measured. Three samples for each extract, obtained by independent experiments, were twice analyzed, and the means of the data were statistically treated (as described below) to assess the significant differences against the control values.

### 2.4. Determination of Antioxidant Activity (DPPH Assay)

The antioxidant activity was evaluated by the DPPH radical scavenging activity [13, 14]. DPPH radical solution (0.002%, w/v) in methanol was prepared, and a volume of 1800 *μ*L was added to 200 *μ*L of the sample diluted with phosphate buffer (0.1 M), well vortexed, and incubated for 60 min in dark room at room temperature. The absorbance of each sample at 520 nm was measured using Shimadzu UV-VIS double-beam spectrophotometer. Methanol was used as a blank, while DPPH solution in methanol served as control. The antioxidant activity was expressed as the percentage inhibition of DPPH radical. The experiments were conducted in triplicate, and the mean values were used.

### 2.5. Microbial Enumeration and Isolation

A 10^–1^ dilution of Laban was made using physiological saline, and the microbial counts were determined according to the pour plate method of Houghtby et al. [15]. Total viable counts were determined using plate count agar (Scharlau Chemie S.A., Barcelona, Spain) incubated at 32°C for 48 h. Counts of lactic acid bacteria (LAB) were determined using de Man Rogosa Sharpe (MRS, Himedia) agar incubated anaerobically at 35°C for 48 h [16], while the count of *Streptococcus* strains was determined using M17 agar (Himedia) incubated anaerobically at 37°C for 48 h. Identification of bacterial colonies from the agar plates was performed by Gram staining, cell morphology, carbohydrate fermentation tests, sensitivity to different salt levels, and catalase reaction [17]. The counts were expressed as log 10 cfu/ml of the product.

## 3. Results and Discussion

The study included 57 children made up of 54 (94.73%) males and 3 (5.26%) females with a mean age of 10.07 years. Male children showed higher G6PD deficiency prevalence rates than females. These results are in accordance with different previous research studies that indicated that hemolysis induced by G6PD deficiency is most common in hemizygous males compared to homozygous females as the mutation would have to occur in both copies of the gene in females to cause the disorder, whereas in males only one abnormal copy of the gene is required for manifestation of the disease [18, 19].

Table 1 shows the distribution of G6PD deficiency using the categorization of the enzyme function into severe deficiency (<10% G6PD activity), moderate deficiency (10–30%), mild deficiency (30–60%), and normal activity (60–100%) [20]. Of the 57 recruited children, twenty-one (36.84%) were moderately deficient, while eighteen (31.57%) were mildly deficient.

Red blood cells (RBCs) contain many antioxidant components, and the G6PD enzyme is essential for maintaining the NADPH supply, which is the key source of reducing equivalents such as glutathione reductase (GSH) and catalase [21]. Therefore, NADPH production by G6PD is critically important, and when it becomes limited, red cells are prone to being damaged [22]. Both vicine and convicine present in fava beans are converted in the gut into divicine and isouramil which are highly redox proteins identified as the main factors of favism [23]. These molecules produce reactive oxygen species (ROS) including the superoxide anion and hydrogen peroxide, which rapidly oxidize NADPH and glutathione. Individuals affected by G6PD deficiency are unable to regenerate reduced glutathione and are undefended against oxidative stress [21]. Acute hemolysis caused by fava bean ingestion is described as being the best-studied natural model of oxidative damage [21]. As presented in Table 1, the incubation of blood samples with fava beans extract (FBE) caused an oxidative RBC damage and hemolysis. The percentage of this hemolysis is correlated with the G6PD deficiency levels as noticed in Table 1. Getachew et al. previously demonstrated that fava bean aglycones cause an irreversible rapid GSH depletion with a simultaneous increase in oxidized glutathione (GSSG) production in G6PD enzyme defective human RBCs [24]. This was correlated with progressive accumulation of denatured hemoglobin products into high molecular weight (HMW) proteins and the formation of both band 3 membrane proteins and hemichromes as HMW protein aggregates [23, 24].

In the most severe type of favism, therapeutic measurements include splenectomy, blood transfusion, food supplements such as folic acid, and antioxidants including vitamin E and selenium [25, 26]. However, favism incidence can be reduced by the decrease of both vicine and convicine from fava beans. Different processing methods such as heat treatment and continuous flow soaking have been studied and proved to achieve a reduction in the content of both glycosides [6, 27]. *β*-Glycosidase from *Aspergillus oryzae*, *Fusarium graminearum*, and lactic acid bacteria was used in bioprocessing methods for a selective destruction of the pyrimidine glycosides [1].

**Figure 1:**
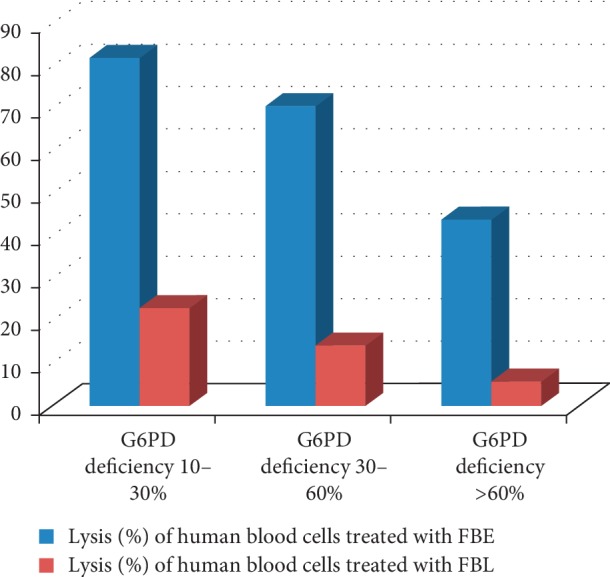
Hemolysis of human blood cells treated with fava bean extract (FBE) and fava beans treated by Laban (FBL).

In the condition of our study, a significant decrease of human blood cell hemolysis was observed after the treatment of fava beans by Laban (Table 1). This decrease in hemolysis was also correlated with the G6PD deficiency categorization. The highest hemolysis level (mean: 23.11 ± 0.76%) was observed in samples with G6PD activity between 10 and 30%, while the lowest hemolysis level (mean: 5.75 ± 0.64%) was observed in samples with G6PD activity more than 60%. In this sense and as Laban mechanism of action has yet to be determined, the antioxidant properties of Laban could partially explain its significant effect (*P* < 0.05) in reducing the hemolysis induced by fava beans (Figure 1). DPPH free radical scavenging assay is widely common to investigate the antioxidant capacity of natural compounds [28]. Results proved that bovine fermented milk used in this study had a high antioxidant capacity of 51.61 ± 1.13% expressed by the percentage inhibition of DPPH radical. Our results are in accordance with Dina Tri et al. [29] and showed a relatively higher value than the value obtained by Fitrotin et al. [30] in the fermentation of sesame milk using L. plantarum Dad 13. This antioxidant capacity is mainly due to peptides released upon proteolysis during the fermentation period and the wide range of metabolic compounds formed by lactic acid bacteria which may act as electron donors reacting with free radicals to form more stable products [31, 32]. Additionally, reductones formed during fermentation could react with free radicals to stabilize and terminate radical chain reactions [33]. This could explain different results previously obtained for the antioxidant properties of fermented milk of different sources which greatly depend on several factors including the food matrix composition, overall peptidic profile, amino acid composition of the individual peptide, and the large differences between species or even strains used in fermentation [34].

It was previously shown that lactobacillus decreases the concentration of vicine and convicine proportionately with the *β*-glycosidase enzyme concentration and the period of treatment [3]. The types of LAB usually found in the fermented dairy products are thermophilic and mesophilic strains of *Streptococcus*, *Lactococcus*, and *Lactobacillus* species [35]. In this study, colonies observed on MRS plates were large and irregular in shape. They had a light color with opaque centers. They were Gram-positive bacteria able to produce acid from lactose, glucose, and fructose. As a result, characteristics of these strains suggested that they belong to *Lactobacillus delbrueckii* subsp. bulgaricus species according to the criteria given by Teixeira [36]. Colonies observed on M17 plates were smaller and regular. They were Gram-positive and spherical bacteria resistant to NaCl (2%), but sensitive to higher salt concentrations. These results indicated that the isolates belonged to the *Streptococcus thermophilus* species [37]. The counts of isolates from MRS and M17 culture plates were 6.75 ± 0.095 and 7.91 ± 0.061 log cfu ml^–1^, respectively. Similar results of total bacteria count were reported for different traditional dairy products [16, 38–40].

## 4. Conclusion and Further Prospective

In conclusion, the synergy between the antioxidant properties of Laban approved by the radical antiscavenging activity and the possible decrease of vicine and convicine concentrations by lactobacillus found in the fermented dairy products could explain the ability of Laban to reduce the hemolysis crisis *ex vivo*. This decrease in hemolysis was correlated with the G6PD deficiency categorization.

Moreover, lactobacillus was previously proved to increase the amount of antioxidant compounds through intestine which would have a more protecting effect against the hemolysis crisis. However, both vicine and convicine are converted in the gut into divicine and isouramil which are highly redox proteins identified as the main factors of favism. So, more research studies should be conducted in order to prove the potential synergistic effect between Laban and fava beans *in vivo*.

## Figures and Tables

**Table 1:** Prevalence of G6PD deficiency levels and hemolysis of human blood cells treated with FBE and FBL.

G6PD deficiency categorization	Prevalence of G6PD deficiency levels	Lysis (%) of human blood cells treated with FBE	Lysis (%) of human blood cells treated with FBL
	Total samples tested 57 (100%)	—	

<10% (<0.64 U/gHb)	0	—	

10–30% (0.64–1.92 U/gHb)	21 (36.84%)	70.74 ± 1.26%–90.65 ± 2.09% Mean: 82.06 ± 1.63%	19.95 ± 0.95%–26.63 ± 0.68% Mean: 23.11 ± 0.76%

30–60% (1.92–3.84 U/gHb)	18 (31.57%)	67.13 ± 1.65%–76.09 ± 1.96% Mean: 70.61 ± 1.36%	9.68 ± 1.35%–21.48 ± 0.96% Mean: 14.13 ± 1.05%

>60% (>3.84 U/gHb)	18 (31.57%)	35.80 ± 2.06%–47.85 ± 1.05% Mean: 44.06 ± 1.49%	1.21 ± 0.23%–9.71 ± 0.68% Mean: 5.75 ± 0.64%

## Data Availability

The data used to support the findings of this study are available from the corresponding author upon request.
